# Effects of sevoflurane and clonidine on acid base status and long-term emotional and cognitive outcomes in spontaneously breathing rat pups

**DOI:** 10.1371/journal.pone.0173969

**Published:** 2017-03-20

**Authors:** Nicole Almenrader, Paola Colucci, Valentina De Castro, Daniela Valeri, Maura Palmery, Viviana Trezza, Patrizia Campolongo

**Affiliations:** 1 Department of Physiology and Pharmacology, Sapienza University of Rome, Rome, Italy; 2 Department of Anaesthesia and Intensive Care, Policlinico Umberto I, Rome, Italy; 3 Department of Science, Section of Biomedical Sciences and Technologies, University “Roma Tre”, Rome, Italy; University of L'Aquila, ITALY

## Abstract

**Background:**

Numerous experiments in rodents suggest a causative link between exposure to general anaesthetics during brain growth spurt and poor long-lasting neurological outcomes. Many of these studies have been questioned with regard of their translational value, mainly because of extremely long anaesthesia exposure. Therefore, the aim of the present study was to assess the impact of a short sevoflurane anaesthesia, alone or combined with clonidine treatment, on respiratory function in spontaneously breathing rat pups and overall effects on long-lasting emotional and cognitive functions.

**Methods:**

At postnatal day (PND) 7, male Sprague Dawley rat pups were randomized into four groups and exposed to sevoflurane for one hour, to a single dose of intraperitoneal clonidine or to a combination of both and compared to a control group. Blood gas analysis was performed at the end of sevoflurane anaesthesia and after 60 minutes from clonidine or saline injection. Emotional and cognitive outcomes were evaluated in different group of animals at infancy (PND12), adolescence (PND 30–40) and adulthood (PND 70–90).

**Results:**

Rat pups exposed to either sevoflurane or to a combination of sevoflurane and clonidine developed severe hypercapnic acidosis, but maintained normal arterial oxygenation. Emotional and cognitive outcomes were not found altered in any of the behavioural task used either at infancy, adolescence or adulthood.

**Conclusions:**

Sixty minutes of sevoflurane anaesthesia in newborn rats, either alone or combined with clonidine, caused severe hypercapnic acidosis in spontaneously breathing rat pups, but was devoid of long-term behavioural dysfunctions in the present setting.

## Introduction

Multiple studies have raised concerns regarding direct neurotoxic effects of general anaesthetics on the developing brain with possible long lasting impact on neurocognitive function [1]. Alterations, such as attention deficit/hyperactivity disorders (ADHD) [2], learning deficits [3], behavioural disturbances [4] and language deficits [5] have been reported in retrospective cohort follow up studies in children. Time of exposure as well as the cumulative anaesthetic dose have both been identified as determinant factors of neurotoxicity. There is clear evidence from animal studies that the time of synaptogenesis is the most vulnerable period for anaesthetic neurotoxicity. Different brain regions are vulnerable at different time windows. In rats, extensive synaptogenesis takes place in the thalamus, hippocampus and neocortex at postnatal day (PND) 7 [6]. Therefore, most studies in this field have been performed by exposing rodents to general anaesthetics at this specific time frame. A recent study in young rodents suggested that physiological disturbances during anaesthesia might contribute to volatile anaesthetic induced neurotoxicity [7]. A higher overall mortality as well as a significant increase in hippocampal cell death and worse performance in the Morris water maze test were reported in spontaneously breathing, anaesthetized rats compared to those who were mechanically ventilated or control group animals.

Stratmann and coworkers observed a similar pattern of neuronal cell death in rat pups exposed to either 4h of isoflurane or 4h of hypercarbia suggesting that part of isoflurane induced cell death might be due to isoflurane induced hypercarbia [8]. However these findings did not translate consistently into neurocognitive outcome as only those animals treated with 4 h of isoflurane anaesthesia showed long-term neurocognitive deficits.

The impact of hypercapnic acidosis on neurocognitive outcome has also been questioned in neonates undergoing thoracoscopic surgery for repair of congenital diaphragmatic hernia and oesophageal fistula [9,10].

Interestingly, the European paediatric anaesthesia community is currently leading an active debate on how to define ‘safe conduct of anaesthesia’ in neonates and young children, pointing out the primary importance of controlling and maintaining stability of physiological parameters [11]. So the current question is, if it is the anaesthetic agent itself or rather the anaesthesia induced derangement of body homeostasis having detrimental effects on the developing brain [12,13].

In the search of preventive and protective strategies, alpha-2-agonists gained an important role being attributed with neuroprotective properties attenuating the apoptotic and neurodegenerative effects of NMDA-antagonists and GABA_A_-agonists [14–16]. The use of clonidine in paediatric anaesthesia is expanding as a result of its’ sedative and analgesic properties [17–19]. On this background we tested the hypothesis that clonidine and 1h of 2.5% sevoflurane, either alone or in combination, would differently affect the respiratory system in spontaneously breathing animals. In order to evaluate if early exposure to anaesthesia—induced hypercarbia could lead to long-lasting behavioural alterations, emotional and cognitive functions were assessed as secondary outcomes not only at infancy (PND12), but also at adolescence (PND 30–40) and adulthood (PND 70–90).

## Materials and methods

### Animals

All animal experiments were approved by the Italian Ministry of Health (Rome, Italy) and performed according to the guidelines of the Italian Ministry of Health (D.L. 96/1992) and the European Community Directive 2010/63/EU of September 22^nd^ 2010.

Multiparous pregnant female Sprague-Dawley rats (Charles River®, Calco, Italy) were housed in an air-conditioned room (temperature 21±1°C) in a standard 12-h light-dark cycle (lights on at 06:00 h), with access to food and water ad libitum.

Newborn litters, found up to 5 pm, were considered to be born on that day (PND0). On PND1 all litters were reduced to a size of 6 males and 2 females per litter as previously described [20].

On PND21, pups were weaned and housed in groups of three in 42 × 27 × 14 cm Plexiglas cages in air-conditioned rooms. One male pup per litter from different litters per treatment group was used in each experiment. Each male rat was tested only once. Females were not tested.

### Randomization

At PND7 rats were randomized by a computer generated list (Graph Pad Software, Inc., California, USA) into four groups receiving: sevoflurane (group S), clonidine (group C), sevoflurane/clonidine (group SC), saline (control group).

### Anaesthesia protocol

Animals anaesthetized with sevoflurane (Sevorane, Abbott, Italy) were placed in a heated anaesthesia chamber connected to a vaporizer system and to a gas anaesthesia machines (Fluovac System, Harvard Apparatus, Holliston, US) breathing sevoflurane 2.5% in oxygen and air (1:1) with a fresh gas flow of 2 l/min. Animals treated with clonidine (Catapresan 150 mcg/ml, Böhringer Ingelheim, Italy) received an intraperitoneal injection of 400 μg/kg diluted in sterile saline to a standard volume of 200 l immediately before being placed in the anaesthetic chamber. Control animals and those exposed to sevoflurane received an intraperitoneal administration of 200 l of sterile saline. The duration of stay in the gas chamber was set to a total time of 60 minutes for comparison with sevoflurane exposure. The chamber was heated and the temperature maintained constant by an infrared light and a heating pad. The concentration and duration of sevoflurane administration were established after preliminary results and previous published studies on rat pups. We decided to omit anaesthesia induction with a high concentration of sevoflurane in order to minimize cardiorespiratory depressant effects. Anaesthesia induction was defined as time from the beginning of sevoflurane until loss of righting reflex. The clonidine dose was chosen according to Kesavan and coworkers [21]. Arterial pulse oximetry and heart rate (MouseOx Plus®, Starr Life Sciences Corp., Oakmont, US) were recorded continuously in one animal every set of four animals undergoing anaesthesia. All other animals were clinically observed for any sign of distress during the entire procedure.

### Blood gas analysis

Blood samples for blood gas analysis were taken from the left ventricle after 60 minutes of drug or saline administration or after 60 minutes of placement in the gas chamber. Arterial blood was analysed immediately after blood collection with a blood gas analyser (GEM Premier 4000, Instrumentation Laboratory SpA, Milan, Italy). After blood sampling animals were sacrificed by cervical dislocation.

### Behavioural testing

The series of behavioural tests was chosen on the basis of the relatively well established anatomical areas involved in these tasks. All behavioural tests were performed by the same operator, who was blinded to group assignment. Adolescent and adult rats were handled three times for one minute each day, starting 72 hours before testing. Emotional and cognitive assessments were performed at infancy (PND12), adolescence (PND30-40) and at adulthood (PND70-90).

### Behavioural tests at infancy

#### Ultrasonic vocalization (USV)

The ultrasonic vocalization (USV) analysis is a useful tool to assess pup social communication skills in the first few days of life [22]. The USV test has previously been studied to assess autism-like behaviour in rodents after neonatal exposure to sevoflurane [23]. When separated from the mother and the nest, newborn rats emit USVs with frequencies between 30 and 90 kHz. These USVs are essential for the mother/pup interaction and are critical for pups survival [22]. At PND 12, pups were removed from the nest and placed in a soundproof arena (30 × 30 × 30 cm). The recording session lasted 3 min [20]. USV calls were recorded by the Avisoft recorder (Avisoft Bioacoustics, Berlin, Germany).

#### Homing test

One of the earliest expressions of spatial behaviour is the pups’ ability to return towards their nest if separated from mother and littermates. Hippocampal formation plays a pivotal role in spatial cognition and navigation [24]. The homing test is a useful task for the evaluation of these rudimentary cognitive abilities in infant rats [25]. At PND 12, each pup was placed in a Plexiglas arena (36×22.5×10 cm) with the bottom covered by 2/3 clean litter and by 1/3 of litter from the mother’s cage, with the nest odour, representing the nest area. The pup was placed close to the wall on the clean litter side and video-recorded for 4 min. The latency to reach the nest area and the time spent in the nest area were recorded to evaluate homing performance.

### Behavioural tests at adolescence and adulthood

#### Inhibitory avoidance

The inhibitory avoidance test is a commonly used task to investigate learning and long-term memory processes in rodents [26]. This test has been used previously to measure sevoflurane-induced impairment of memory consolidation in rodents [27]. The procedure was carried out as previously described by Morena [28]. For training, adolescent and adult rats were placed into the starting compartment and were allowed to explore the apparatus. After the rats stepped into the dark compartment, the sliding door was closed, and a single footshock (0.4 mA) was delivered for 1 s. Animals were removed from the shock compartment 15 s after termination of the footshock. Retention was tested 48 h later. On the retention test, rats were placed into the starting compartment, and the latency to re-enter the shock compartment (maximum latency of 600 s) was recorded. Longer latencies were interpreted as indicators of better retention.

#### Elevated plus maze

The elevated plus maze (EPM) is a validated assay to study hippocampus dependent behaviour performance in rodents [29] and has been applied previously to assess anxiety related behaviour in rodents after inhalational [30] and intravenous anaesthetics [31]. The EPM apparatus comprised two open arms (50 × 10 × 40 cm) and two closed arms (50 × 10 × 40 cm) that extended from a common central platform (10 × 10 cm). Confinement to the closed arms was associated with the observation of significantly more anxiety-related behaviours than confinement to the open arms [32]. The EPM test was performed following the procedure described by Manduca and colleagues [33]. Adolescent and adult rats were individually placed on the central platform facing a closed arm and a 5-min test period was recorded. Behavioural analysis was carried out using the Observer® XT 13.0 software (Noldus, Wageningen, The Netherlands). The following parameters were analysed:

- % Time spent on the open arms (% TO);
- % Open entries (% OE);
- Number of exploratory head dippings (HDIPS);
- Number of stretched-attend postures (SAP).

#### Statistical analysis

Data are expressed as mean ± standard error of mean (SEM). To assess the effects of different treatment, data were analysed using one-way ANOVA (Graph Pad Prism version 6.0) followed by Tukey’s multiple comparison post-hoc test where appropriate. For all comparisons, p values of less than 0.05 where considered statistically significant.

## Results

There were no differences in demographic data between groups (Table 1). Mean time and SEM of anaesthesia induction was 4.8 ± 0.5 minutes in the sevoflurane group and 4.1 ± 0.3 minutes in the sevoflurane/clonidine group (p = 0.3). There was no anaesthesia related mortality.

**Table 1:** Demographic data.

	CONTROL	CLONIDINE	SEVOFLURANE	SEVOFLURANE/ CLONIDINE

**BW (g)**	17.90 ± 0.78	16.89 ± 0.72	16.30 ± 0.79	17.85 ± 0.80
**T°C pre- treatment**	36.70 ± 0.08	36.63 ± 0.18	36.68 ± 0.11	36.68 ± 0.08
**T°C post- treatment**	36.71 ± 0.08	36.64 ± 0.17	36.93 ± 0.05	36.89 ± 0.05

Data are presented as mean ± standard error of mean (SEM). There was no difference of body weight and temperature among groups (n = 10 per group; p > 0.05). BW = body weight. T°C = axillary body temperature.

### Blood gas analysis

Mean and SEM values for all parameters are reported in Table 2.

**Table 2:** Blood gas analysis.

	CONTROL	CLONIDINE	SEVOFLURANE	SEVOFLURANE/CLONDINE
**pH**	7.39 ± 0.01	7.39 ± 0.01	7.25 ± 0.04*	6.99 ± 0.04*
**pCO**_**2**_ **mmHg**	47.50 ± 1.53	50.70 ± 2.10	81.40 ± 7.81*	133.50 ± 9.70*
**lactate mmol/L**	3.55 ± 0.25	2.68 ± 0.14*	1.10 ± 0.13*	0.66 ± 0.11*
**HCO**_**3**_ **mmol/L**	26.39 ± 0.57	27.84 ± 0.58	27.79 ± 0.65	26.45 ± 1.66
**BE mmol/L**	3.89 ± 0.61	5.57 ± 0.83	7.25 ± 1.2	6.13 ± 1.37
**glucose mg/dL**	113 ± 4.27	141 ± 7.77	127 ± 7.82	112 ± 16.36
**pO**_**2**_ **mmHg**	93.40 ± 2.71	92.70 ± 2.68	88.30 ± 1.48	90.13 ± 1.7

Values are reported as mean ± standard error of mean (SEM).

* p<0.05. BE = base excess, pH, pCO_2_ and lactate levels were significantly lower in rat pups treated with sevoflurane or sevoflurane and clonidine. There were no differences among groups for bicarbonate, pO_2_ and glucose levels (p > 0.05).

#### pH and pCO_2_

Rat pups treated with either sevoflurane or a combination of sevoflurane and clonidine developed severe respiratory acidosis, while there was no difference in pH and pCO2 between control animals and those receiving clonidine (F _(3,34)_ = 46.82, *P* < 0.0001; F _(3,34)_ = 41.16, *P* < 0.0001 for pH and pCO_2,_ respectively).

#### Lactate

Lactate levels were significantly lower in animals treated with sevoflurane alone or in combination with clonidine compared to control and clonidine treated rats. Lactate levels of rat pups treated with clonidine alone were significantly lower compared to the control group (F _(3,34)_ = 61.35; *P* < 0.0001).

#### HCO_3_

One-way ANOVA did not reveal any significant difference in bicarbonate levels among groups (F _(3,34)_ = 0.8338; *P* = 0.48)

#### pO_2_

No cases of hypoxia were observed. There was no statistically significant difference in pO_2_ between groups (F _(3,34)_ = 1.115; *P* = 0.36)

#### Base excess

One-way ANOVA did not show any significant difference among groups (F _(3,34)_ = 2.004; *P* = 0.13)

#### Glucose levels

No significant differences were observed among groups (F _(3,34)_ = 2.205; *P* = 0.10)

### Behavioural tests

#### USV and homing test

No significant effects of treatment on USVs were observed (F _(3, 38)_ = 0.91, *P* = 0.45, Table 3). As concerning the homing test, one-way ANOVA did not reveal any significant effect of treatment on the latency time (F _(3, 38)_ = 0.85, *P* = 0.48) and on the nesting time (F _(3, 38)_ = 0.44, *P* = 0.73) in the homing test (Table 3).

**Table 3:** Ultrasound vocalization (USV) and homing test at infancy.

		CONTROL	CLONIDINE	SEVOFLURANE	SEVOFLURANE/ CLONIDINE
**USV**	**Frequency (n)**	691.08 ± 100.37	619.20 ± 104.39	448.80 ± 123.71	624.10 ± 107.17
**HOMING TEST**	**Latency (s)**	83.34 ± 23.25	91.34 ± 28.53	82.50 ± 27.27	136.95 ± 32.51
	**Nesting time (s)**	145.42 ± 21.39	152.25 ± 21.37	190.49 ± 23.78	115.90 ± 27.48

Data are expressed as mean ± standard error of mean. There were no differences of behavioural tests among groups (n = 10 per group; p > 0.05). s = seconds; n = numbers of vocalizations

#### Inhibitory avoidance

No significant differences were found in the inhibitory avoidance test among groups either at adolescence or adulthood respectively (training: F _(3, 28)_ = 0.55, *P* = 0.65; test: F _(3, 28)_ = 1.01, *P* = 0.40; training: F _(3, 38)_ = 0.44, *P* = 0.72; test: F _(3, 38)_ = 0.61, *P* = 0.61, Table 4).

**Table 4:** Inhibitory avoidance test (IA) at adolescence and adulthood.

		CONTROL	CLONIDINE	SEVOFLURANE	SEVOFLURANE/ CLONIDINE
**ADOLESCENCE**	**Training Latency Time (s)**	9.07 ± 1.75	8.13 ± 1.31	8.29 ± 2.36	6.06 ± 1.25
	**Test Latency Time (s)**	140.42 ± 71.30	352.95 ± 97.78	285.52 ± 89.43	294.36 ± 98.42
**ADULTHOOD**	**Training Latency Time (s)**	10.03 ± 1.41	10.83 ± 2.10	12.66 ± 2.62	9.83 ± 1.41
	**Test Latency Time (s)**	352.44 ± 74.76	295.91 ± 59.13	356.48 ± 69.81	239.12 ± 73.12

Latency times are measured in seconds (s). Results are expressed as mean ± standard error of mean (SEM). There was no difference among groups (n = 10 per group; p > 0.05).

#### Elevated plus maze

**Fig 1:**
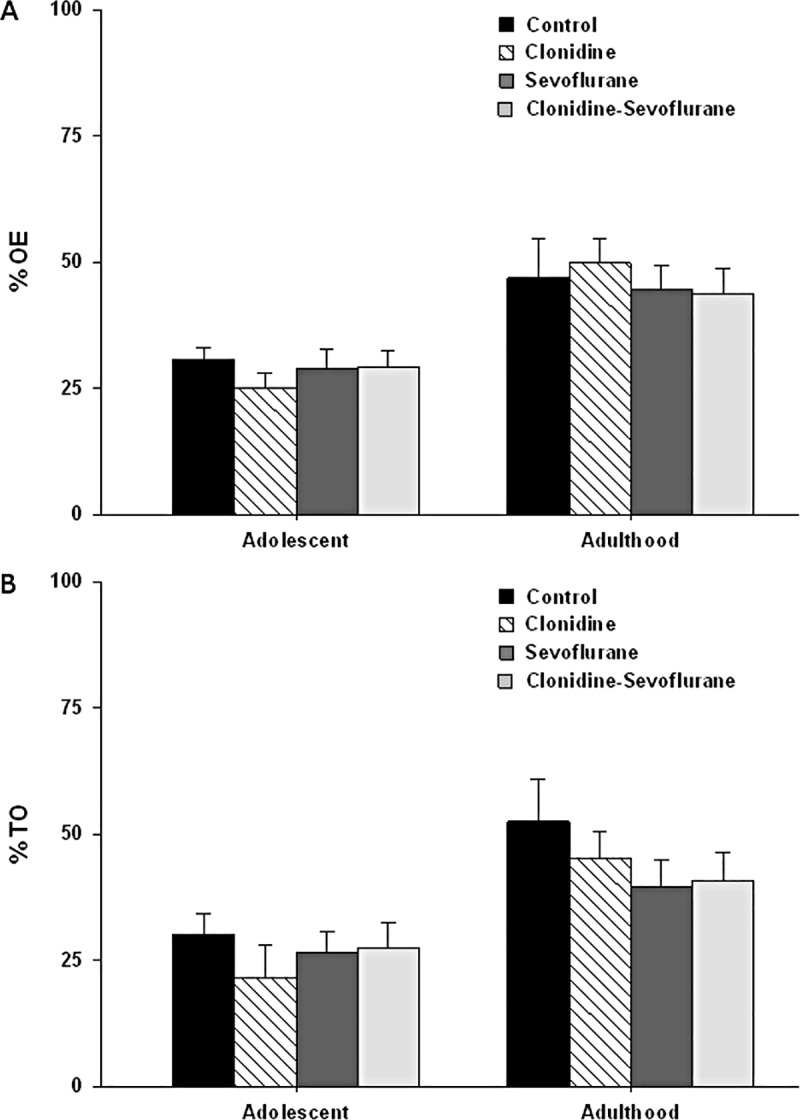
**(A): Elevated Plus Maze (EPM) at adolescence and adulthood.** Percentage of entries in the open arms (OE). Results are expressed as mean ± standard error of mean (SEM). There was no difference among groups (n = 10 per group; p > 0.05). (**B): Elevated Plus Maze (EPM) at adolescence and adulthood** Percentage of time spent in the open arms (TO). Results are expressed as mean ± standard error of mean (SEM). There was no difference among groups (n = 10 per group; p > 0.05).

One-way ANOVA showed that there was no significant effect of treatment at adolescence on %TO (F_(3, 38)_ = 0.55, *P* = 0.65; Fig 1A), on %OE (F_(3, 38)_ = 0.62, *P* = 0.60; Fig 1B), on HDIPS frequency (F_(3, 38)_ = 0.74, *P* = 0.54) and on SAP Frequency (F_(3, 38)_ = 0.55, *P* = 0.65) and at adulthood on: %TO (F_(3,34)_ = 0.89, *P* = 0.46; Fig 1A), %OE (F_(3,34)_ = 0.27, *P* = 0.85; Fig 1B), in HDIPS frequency (F_(3,34)_ = 1.51, *P* = 0.23), and in SAP frequency (F_(3,34)_ = 1.25, *P* = 0.31).

## Discussion

To date there is an open debate on whether impaired long-term neurocognitive outcome after anaesthesia in newborn rodents and humans might be a result of a direct drug related neurotoxic effect or rather a consequence of physiological disturbances occurring during anaesthesia [7,11,34].

The present findings show that even a relatively brief anaesthesia with sevoflurane causes profound hypercapnic acidosis (HCA) in spontaneously breathing rat pups. This effect is aggravated by the contemporary administration of clonidine. However, no correlation could be found between anaesthesia–induced hypercapnic acidosis at PND 7 and emotional or cognitive alterations at infancy, adolescence and adulthood in any of the behavioural tasks used in the current study. HCA has been suggested as a determinant factor in anaesthesia related neurotoxicity and long-term cognitive outcome [7,8].

Stratmann and coworkers showed that neonatal exposure to either two and four hours of isoflurane or four hours of carbon dioxide caused a similar pattern and distribution of cell death in the cortex, hippocampus and thalamus [8]. However long term neurocognitive deficits were only found in rodents after four hours of isoflurane exposure suggesting a dose-dependent threshold.

In contrast Wu and co-workers [7] compared spontaneously breathing to mechanically ventilated 14-day old rats that were exposed to four hours of either sevoflurane or isoflurane. Self—ventilating animals showed a higher percentage of overall mortality (30%), neuronal cell death and alterations of spatial learning compared to mechanically ventilated and control animals. HCA has important biochemical and physiological effects [35].

The effect of HCA on brain function in neonates is of uttermost interest among neonatologist when applying protective ventilation strategies with permissive hypercapnia. Extremes of hypercapnia and especially rapid changes of PaCO_2_ have been linked to adverse neurological outcome in premature neonates [36]. Furthermore, HCA was shown to inhibit neuronal metabolism and to increase expression of pro-apoptotic mediators in the cerebral cortex of newborn piglets [37]. Notwithstanding the above concerns, several animal studies have suggested neuroprotective effects of hypercapnia on impaired neuronal function in the injured neonatal rat brain [38–40].

Vannucci and coworkers showed that hypercapnia protects the immature rat brain from hypoxic—ischaemic damage. Improved cerebrovascular perfusion and oxygen delivery as well as enhanced cerebral glucose utilization were observed in animals exposed to hypoxia and hypercapnia. Besides a marked attenuation in cerebral tissue lactacidosis, a reduction of the excitatory amino acid neurotransmitter glutamate in the CSF was found [38,39].

There has been recently a debate on safety of thoracoscopic surgery for congenital diaphragmatic hernia (CDH) and oesophageal atresia in the newborn [9,10]. A prospective pilot randomized controlled trial at Great Ormond Street Hospital provided evidence that thoracoscopy in neonates is associated with significant hypercapnia and acidosis. The levels of hypercapnia and acidosis were of such concern that thoracoscopic repair of CDH in this age group was suspended. Six out of 10 neonates experienced paCO_2_ levels above 105 mmHg for a duration ranging from 30 minutes up to three and a half hours. There were no changes of arterial oxygenation. Results from neurodevelopmental follow-up of these kids are still awaited and might elucidate long-term effects of hypercapnia and acidosis on the developing brain [10]. There are no reports in the literature as yet indicating a deleterious cerebral effect of acidosis and hypercapnia in the presence of normoxia.

A case report of an 8—year old, asthmatic boy suffering more than 14 hours of severe hypercapnic acidosis due to respiratory failure was followed by complete recovery [41]. A second case of severe hypercapnia under sevoflurane anaesthesia was reported in a three—week old baby undergoing incarcerated hernia repair. No neurological sequelae were observed [42]. A reduction in cerebral metabolic requirements due to deep sedation with sevoflurane as well as adequate tissue perfusion and oxygenation were proposed as protective mechanisms for tolerating severe hypercapnia.

Volatile anaesthetic preconditioning (APC) of the brain has been demonstrated both, in vitro and in vivo, yet the underlying mechanism is not fully understood. Suppression of energy requirements seems not sufficient to explain the neuroprotective effects of sevoflurane. Further mechanisms such as inhibition of glutamate release, direct scavenging of free radicals, anti-oxidative and anti-inflammatory effects as well as prevention of apoptotic cell death seem to play an important role for increasing cerebral ischaemic tolerance [43].

In the present study neonatal rodents were exposed to sevoflurane and as a consequence of anaesthesia induced respiratory depression also to hypercapnic acidosis. However, oxygenation and tissue perfusion were guaranteed as shown by normal serum paO_2_ and lactate levels. The battery of neurocognitive tests utilized focused on the detection of more subtle changes related to social communication, autism-like behaviour and anxiety.

More recent studies in neonatal rodents assessing autism-like behaviour, social behaviour and fear conditioning after sevoflurane exposure [23,30] reported conflicting results. Noteworthy, most of these experiments used extremely long anaesthesia protocols with up to six hours of exposure to inhalational anaesthetics. When translating time from newborn rat pups to human beings, six hours in a rat pups’ life would have an equivalent of more than two days in human life [44]. Not surprisingly very high mortality rates up to 30%^7^ are reported in these experiments, especially in the final hours of the anaesthesia. A direct correlation between the cumulative dose of general anaesthetics and the risk of neurocognitive impairment has been postulated [1,2,45].

The role of alpha-2-agonists in the context of neuroprotection needs still to be defined. Few studies in rodents have been performed suggesting protective properties [14–16].

Pre- and postconditioning with dexmedetomidine and clonidine has been reported to attenuate cerebral ischaemia–reperfusion injury in rats [46–48].

Conversely, in relation to the possible neuroprotective effects of clonidine [15,16], our findings suggest that clonidine alone has no impact either on respiratory function nor on neurocognitive outcome in rat pups. In combination with sevoflurane a rather synergistic depressant effect on the ventilatory response to carbon dioxide was observed. This is in accordance with findings of Kesavan and coworkers [21] showing a 35% reduction in respiratory rate after 90 minutes of intraperitoneal injection of clonidine 400 mcg/kg in neonatal rats. No conclusions regarding possible neuroprotective effects can be drawn from the present study as the battery of behavioural tests did not reveal any deficit in any group treated. More detailed studies in this field are warranted.

An unexpected finding of our study was the inverse relationship between serum lactate and carbon dioxide. The regulation of serum lactate in rat pups is not fully understood and it has been suggested that lactate may play a pivotal role as substrate of cerebral metabolism during the neonatal period [49]. It has been shown that repeated tactile stimulation of neonatal rats increases serum lactate levels by 207%. However, on PD7 this effect was reduced to 11%. No increase in serum lactate levels was observed in anaesthetized rat pups [50]. This observation is in accordance with our findings showing lower serum lactate levels in anaesthetized rat pups compared to controls.

### Limits of the study

The present study design does not allow a clear separation between anaesthesia and hypercarbia related effects as a hypercarbic (CO_2_) control group is lacking. However, results give insight in an ‘overall’ combined effect of general anaesthesia and hypercapnic acidosis on the developing brain opening the hypothesis that general anaesthesia might be protective under these conditions.

## Conclusions

Results of the present study suggest that a brief exposure to sevoflurane and severe hypercarbia might not affect long-term cognitive and emotional functions during the period of brain growth spurt. However, the absence of behavioural and emotional deficits does not preclude other forms of behavioural impairments, which might have been detected by more specific tasks others than those used in the present study.
